# Intracranial Volume Not Correlated With Severity in Trigonocephaly

**DOI:** 10.1177/10556656211025185

**Published:** 2021-06-17

**Authors:** Otto D. M. Kronig, Sophia A. J. Kronig, Léon N. A. Van Adrichem

**Affiliations:** 1 Department of Plastic and Reconstructive Surgery, University Medical Center Utrecht, the Netherlands

**Keywords:** intracranial volume, papilledema, synostosis, trigonocephaly

## Abstract

**Objectives:**

Severity of trigonocephaly varies and potentially affects intracranial volume (ICV) and intracranial pressure (ICP). The aim of this study is to measure ICV in trigonocephaly patients and compare it to normative data and correlate ICV with the severity of the skull deformity according to UCSQ (Utrecht Cranial Shape Quantifier).

**Design:**

Retrospective study.

**Setting:**

Primary craniofacial center.

**Patients, Participants:**

Nineteen preoperative patients with nonsyndromic trigonocephaly (age ≤12 months).

**Intervention:**

Intracranial volume was measured on preoperative computed tomography (CT) scans by manual segmentation (OsiriX Fondation). Utrecht Cranial Shape Quantifier was used to quantify the severity of the skull deformity. When present, papilledema as sign of elevated ICP was noted.

**Main Outcome Measures(s):**

Measured ICV was compared to Lichtenberg normative cranial volume growth curves, and Pearson correlation coefficient was used to correlate UCSQ with the ICV.

**Results:**

Mean age at CT scan was 6 months (2-11). Mean measured ICV was 842 mL (579-1124). Thirteen of h19 patients (11/15 boys and 2/4 girls) had an ICV between ±2 SD curves of Lichtenberg, 2 of 19 (1/15 boys and 1/4 girls) had an ICV less than −2 SD and 4 of 19 (3/15 boys and 1/4 girls) had an ICV greater than +2 SD. Mean UCSQ severity of trigonocephaly was 2.40 (−622.65 to 1279.75). Correlation between severity and ICV was negligible (r = −0.11). No papilledema was reported.

**Conclusions:**

Measured ICV was within normal ranges for trigonocephaly patients, in both mild and severe cases. No correlation was found between severity of trigonocephaly and ICV.

## Introduction

Trigonocephaly is the morphologic consequence of premature fusion of the metopic suture. Currently, it is the second most frequent type of craniosynostosis with an incidence of 1 case per 5200 newborns (Van der Meulen, 2012). Clinical presentation can vary widely, ranging from metopic ridge to a distinct triangular shape of the forehead, based on timing of metopic closure and its extend (Posnick et al., 1994; Reardon, 2000).

A potential consequence of altered skull shape in patients with trigonocephaly is alteration of intracranial volume (ICV), which can lead to elevated intracranial pressure (ICP). Raised ICP comes to expression as papilledema or optic atrophy found by fundoscopy (Florisson et al., 2010). The most popular theory for the etiology of raised ICP in children with trigonocephaly is the craniocerebral disproportion or volume mismatch theory. According to this theory, cerebral growth and frontal bones are restricted, which results in volume mismatch and raised ICP (Kapp-Simon et al., 2007).

The treatment of choice for trigonocephaly (and craniosynostosis in general) is skull vault surgery. This operation enlarges the ICV in order to prevent or treat raised ICP. During the first years of life, when the growth of the brain is the most rapid, children with craniosynostosis are the most at risk for elevated ICP (Renier et al., 1982). Therefore, surgery is preferably performed in the first live year to reduce the risk of developing raised ICP and generate better cognitive outcomes (Renier et al., 2000).

However, currently there is no general acceptance in literature if trigonocephaly is truly associated with a restricted or larger ICV. Some studies suggest that the growth restriction results in a reduced ICV (Anderson et al., 2004; Sgouros, 2005; Van der Meulen, 2012), while other studies stated that ICV in trigonocephaly patients is elevated above derived normal values of age- and sex-matched children (Gault et al., 1990; McCarthy et al., 1995; Posnick et al., 1995).

Furthermore, little is known about the association between severity of trigonocephaly and ICV. Severity of trigonocephaly can be established by using UCSQ (Utrecht Cranial Shape Quantifier) (Kronig et al., 2020a). Utrecht Cranial Shape Quantifier is an outline-based method of classification and quantification of skull shape deformities (Kronig et al., 2020, Kronig et al., 2021). This method has the advantage of capturing the actual skull shape variation with every 3D diagnostic system that captures the surface of the head. External landmarks are used to extract an outline of the skull shape using computed tomography (CT) scans, resulting in sinusoid curves. Specific and characteristic curves and parameters for trigonocephaly are found.

The aim of this study is to measure ICV in preoperative patients with trigonocephaly and compare these values with age- and sex-matched skull volumes of Lichtenberg’s normal population (Lichtenberg, 1960). Additionally, ICV will be correlated with the severity of trigonocephaly according to UCSQ. Presence of papilledema, as a sign of elevated ICP, was noted.

## Materials and Methods

### Patients

Patients with CT-confirmed nonsyndromic trigonocephaly (age ≤12 months) were included for this retrospective study. The patients were diagnosed at the Erasmus Medical Center, Sophia Children’s Hospital Rotterdam.

To be eligible for inclusion, the CT scan needed to contain the whole skull (full region between the vertex and foramen magnum). Patients with additional synostosis, other craniofacial abnormality or (orbital or cranial) surgery prior to the first available CT scan were excluded.

The CT scans used for the purposes of this study were part of the routine diagnostic evaluation in patients with a suspected craniosynostosis. The slice thickness of the CT scans was maximally 3.00 mm. Additionally, preoperative ophthalmic patient records needed to be available.

The study was approved by the local Medical Ethics Review Committee. The study was deemed a retrospective clinical study and did not require formal research ethics approval under the Medical Research Involving Human Subjects Act.

### Calculating the ICV

**Figure 1.**
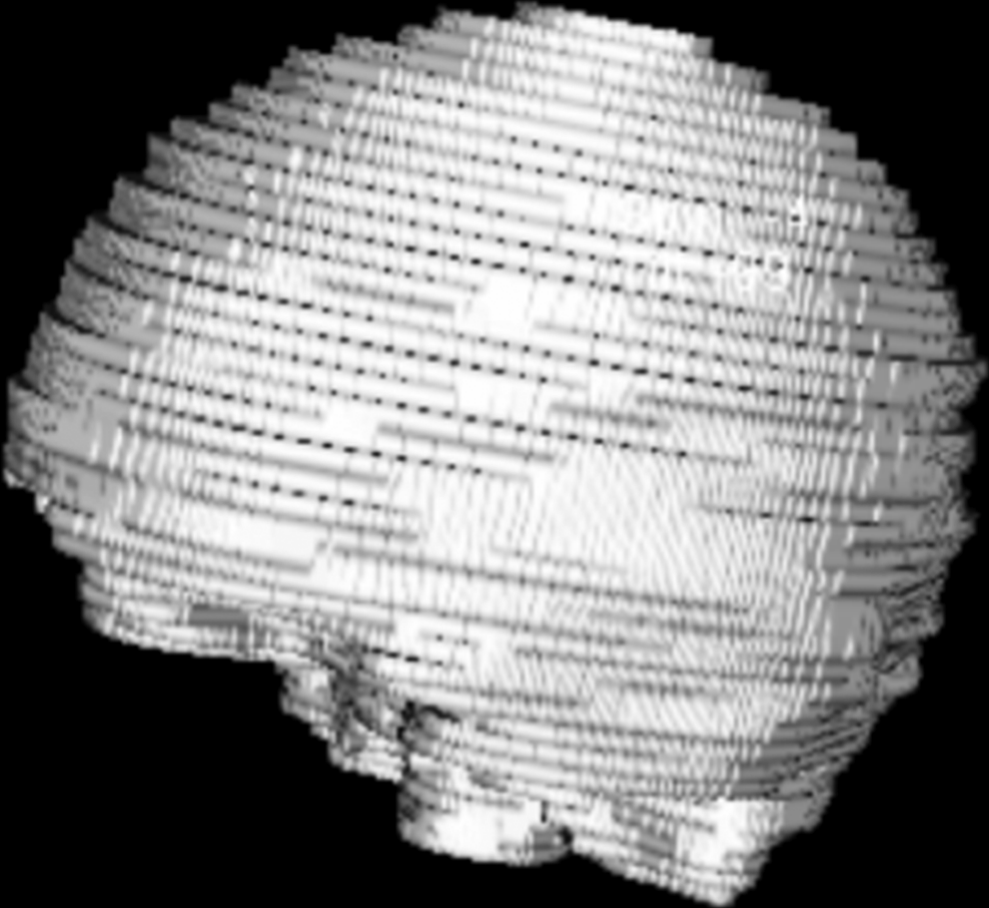
Visualization of calculated and measured intracranial volume (ICV).

The entire intracranial cavity was considered region of interest (ROI) in order to calculate ICV. Computed tomography DICOM images were imported to OsiriX (version 7.0, OsiriX Fondation) on Mac OSX. Start slice was considered just above foramen magnum and end slice just beneath vertex of the skull. On each axial slice, the ROI was manually outlined on the inner table of the skull, defects were manually closed. The total ICV was extrapolated (Figure 1).

### Lichtenberg’s Normal Value of ICV

As reference for normal ICVs of (healthy) children, matched for age and sex, we used Lichtenberg’s data, which is the most frequent reference for normal ICV (Lichtenberg, 1960). There are 5 growth curves: ±2 SD, ±1 SD, and mean.

### Classification of Severity

**Figure 2.**
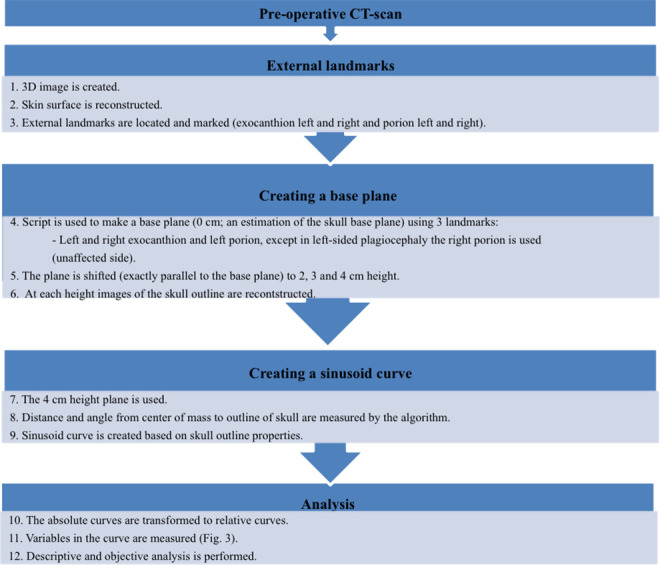
Summary of methods.

The curves, generated by the UCSQ method (Figure 2), were analyzed for different variables. Trigonocephaly can be classified according to severity based on UCSQ for trigonocephaly and its severity calculation (Figure 2) (Kronig et al., 2021).

**Figure 3.**
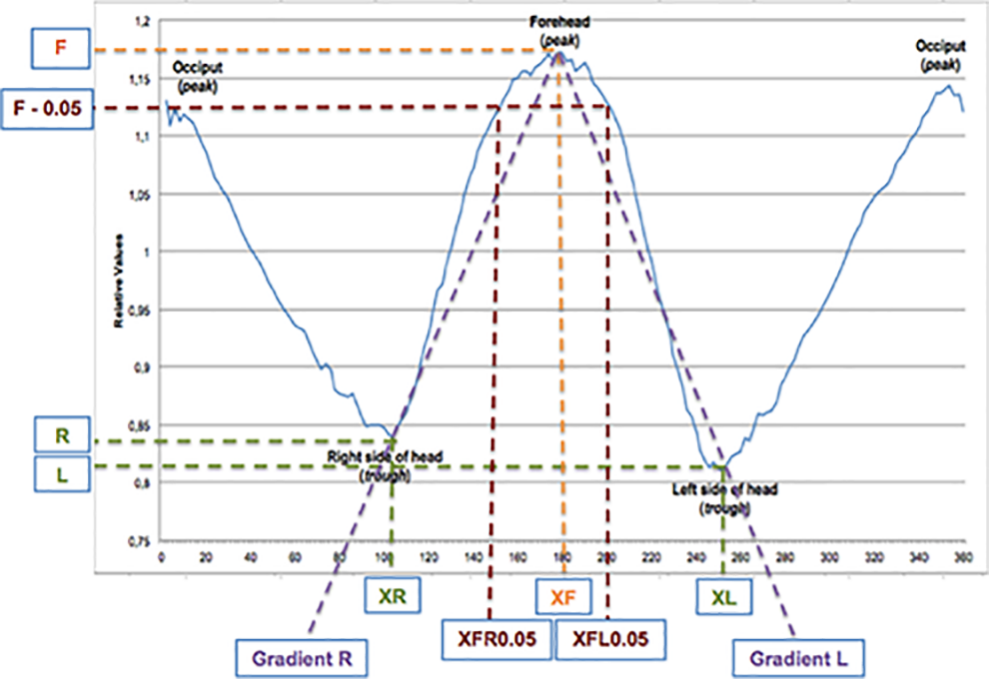
Visualization of the used variables.

Utrecht Cranial Shape Quantifier for trigonocephaly consists of the following 2 variables: ΔY peak (difference between maximum value of the forehead and mean of both sides of the head) and width of frontal peak at F-0.05 (Figure 3). In our previous study, we combined ΔY peak and width of frontal peak at F-0.05. A high correlation was found between severity of trigonocephaly and these combined variables (Kronig et al., 2021).

In order to differentiate between the different levels of severity of trigonocephaly, we will use the introduced severity calculation: (Width of frontal peak at F = 0.05-66) × −2.9 + (ΔY peak − 0.26) × 8300. In this calculation, the values 66 and 0.26 are the values for width of frontal peak and ΔY peak in control patients. In the calculation, the differences between the variables in trigonocephaly patients and controls are multiplied (by −2.9, and 8300) in order to give each variable the same weight in the resulting outcome.

The cutoff values of the calculation in order to classify severity are mild <−500, moderate −500 to 0, severe ≥0.

### Papilledema

As a routine procedure for patients with trigonocephaly, each individual underwent fundus examination at the outpatient clinic, prior to surgery. Patient records were assessed for the presence of preoperative papilledema at fundus examination following mydriasis of the pupil, performed by an ophthalmologist. Papilledema was defined as blurring of the margins of the optic disk (Fried et al., 1982).

### Statistical Analysis

Statistical analyses were performed using the Statistical Package for the Social Sciences for Windows (Version 21, SPSS Inc). Descriptive statistics were calculated.

Pearson correlation coefficient or Spearman rank correlation coefficient was used to determine correlation between ICV and UCSQ. The used test was based on normality of data. The accepted guidelines for interpreting the correlation coefficients are +1 indicates a perfect positive linear relationship, −1 indicates a perfect negative linear, and 0 indicates no linear relationship (Ratner, 2009). The outcomes of the correlation coefficient are characterized as negligible correlation (0.00-0.30), low (0.30-0.50), moderate (0.50-0.70), high (0.70-0.90), and very high (0.90-1.00) (Hinkle et al., 2003).

## Results

We included 19 preoperative children with nonsyndromic trigonocephaly. The average age at time of preoperative CT scan was 6 months (2-11 months). This study included 15 boys and 4 girls (79% vs 21%, respectively). Mean age at fundoscopy was 10 months (2-12 months), all prior to surgery.

### Intracranial Volume

Using the preoperative CT scans, ICV measurements were performed. The preoperative ICVs ranged from 579 mL in a 1-month-old child to an ICV of 1124 mL in a 10-month-old child. Mean ICV of all included patients was 842 mL (Table 1).

**Table 1.** Patient Characteristics and Measured Preoperative Intracranial Volume of Trigonocephaly Patients.

	Gender	Age at preoperative CT scan (months)	Preoperative ICV (mL)	UCSQ score	UCSQ severity class^a^	Compared to Lichtenberg’s data
1	Male	10	993	−565	Mild	Mean to −1SD
2	Male	1	579	796	Severe	Mean to +1SD
3	Male	3	698	−621	Mild	Mean to +1SD
4	Female	8	824	−528	Mild	−1SD to −2SD
5	Male	3	762	305	Severe	+1SD to +2SD
6	Female	3	697	−220	Moderate	+1SD to +2SD
7	Male	8	1124	−623	Mild	+1SD to +2SD
8	Male	5	1004	−314	Moderate	>+2SD
9	Male	6	906	159	Severe	Mean to +1SD
10	Male	2	661	−423	Moderate	Mean to +1SD
11	Male	3	814	−365	Moderate	+1SD to +2SD
12	Female	11	827	1280	Severe	<−2SD
13	Male	8	1044	−392	Moderate	Mean to +1SD
14	Male	2	809	620	Severe	>+2SD
15	Male	5	810	−331	Moderate	Mean to −1SD
16	Male	5	659	−514	Mild	<−2SD
17	Male	2	858	952	Severe	>+2SD
18	Female	4	821	316	Severe	+1SD to +2SD
19	Male	10	1118	511	Severe	Mean to +1SD

Abbreviations: CT, computed tomography; F, female; ICV, intracranial volume; M, male; SD, standard deviation; UCSQ, Utrecht Cranial Shape Quantifier.

^a^ Mild <−500, moderate −500 to 0, severe ≥ 0.

Lichtenberg normative data were used for comparison of normal ICV with preoperative ICV of the patients with trigonocephaly. Twelve (80%) of 15 boys and 2 (50%) of 4 girls had volumes at or larger than the Lichtenberg mean. Of these 12 boys, 6 had an ICV between mean and +1 SD Lichtenberg normative curves, 3 boys had an ICV between +1 SD and +2 SD, and 3 boys had an ICV larger than +2 SD. Both the girls had an ICV between +1 and +2 SD.

**Figure 4.**
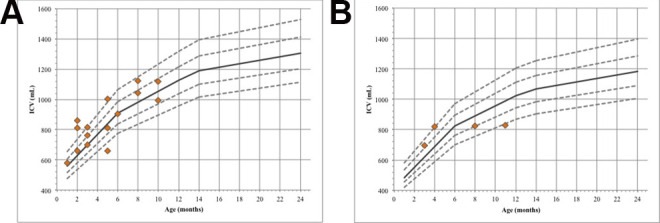
Lichtenberg normative intracranial volume curves are gender- and age-specific curves. Intracranial volume (ICV) measurement of each included patient is plotted on the Lichtenberg normative curves. Orange rhombi indicate patients; dotted lines indicate SD lines (±1 SD and ±2 SD) of Lichtenberg mean; continuous line indicates Lichtenberg mean. (A) Boys (N = 15). (B) Girls (N = 4).

Three (20%) of 15 boys and 2 (50%) of 4 girls had volumes lower than the Lichtenberg mean. Of these 3 boys, 2 had an ICV between mean and −1 SD Lichtenberg normative curves and 1 had an ICV smaller than −2 SD. One of the 2 girls had an ICV between −1 SD and −2 SD and 1 had an ICV smaller than −2 SD. Figure 4A and B shows measurements of ICV of each included patient plotted in these ranges.

In all, 20% (3/15) of the boys had an ICV larger than +2 SD of the mean and 6.7% (1/15) had an ICV lower than −2 SD of the mean. Additionally, 25% (1/4) of the girls had an ICV lower than −2 SD of mean.

### Intracranial Volume and Severity

Used variables for quantification according to UCSQ were ΔY peak (mean 0.3 [0.2-0.4]) and width of frontal peak at F = 0.05 (mean 31.8 [22-44]).

Mean of the calculation for severity of trigonocephaly ([Width of frontal peak at F = 0.05-66] × −2.9 + (ΔY peak − 0.26) × 8300) was 2.40 (−622.65 to 1279.75). Mean (Width of frontal peak at F = 0.05-66) was −34.21 (−44.00 to −22.00), and mean (ΔY peak − 0.26) was −0.01 (−0.09 to 0.14). Based on this calculation and its cutoff values in order to classify the severity of trigonocephaly. According to the cutoff values, 5 patients were categorized as mild, 6 as moderate, and 8 as severe.

### Correlation

Negligible correlation was found between UCSQ and ICV (r = −011).

### Papilledema

Papilledema was not reported in the included patients during fundoscopy.

## Discussion

Since little is known about the correlation between severity of trigonocephaly and its ICV, the present study correlated with the severity of the skull deformity according to UCSQ to ICV. In our previous study, we found high correlation between the UCSQ method and severity of trigonocephaly and is therefore proven to be eligible for quantification purposes in trigonocephaly patients (Kronig et al., 2021).

As stated before, there is discussion concerning ICV in metopic synostosis, due to several controversies in literature. For example, Sgouros et al. (1999) reported that children with single-suture craniosynostosis are born with a restricted ICV, which normalizes by 6 months of age. In 2020, Cronin et al. noted a significantly reduced ICV in a group of 72 children with trigonocephaly compared to healthy children, which normalized at the age of 12 months (Cronin et al., 2020). Additionally, several other studies showed reduced preoperative ICV of children with trigonocephaly (Anderson et al., 2004; Sgouros, 2005). In contrast, various other studies found that ICV in trigonocephaly patients is elevated above derived normal values of age- and sex-matched children (Gault et al., 1990; McCarthy et al., 1995; Posnick et al., 1995).

In the present study, we measured ICV in 19 preoperative patients with trigonocephaly, we compared these volumes by age- and sex-matched skull volumes of Lichtenberg’s normal value (Lichtenberg, 1960). The normative data for skull volumes of healthy children are generally accepted and used by multiple authors (Gault et al., 1990; Fok et al., 1992; Posnick et al., 1995; Abbott et al., 2000; Anderson et al., 2004). We found that 10.5% (2/19) of the trigonocephaly patients of our sample had a smaller (<−2 SD) ICV compared to the mean of the normal values of the group (1/15 boys and 1/4 girls); 68.4% (13/19) of the trigonocephaly patients had a normative (between +2 SD and −2 SD) ICV compared to the normative group (11/15 of the boys and 2/4 of the girls); 21.1% (4/19) of the trigonocephaly patients had a larger (>+2 SD) ICV compared to the normative group (3/15 of the boys and 1/4 of the girls). The overall ICV is within the limits of normal ICV by Lichtenberg; however, we found individuals with both larger and smaller ICV than the mean. Therefore, our findings correspond with literature, stating that both a larger, smaller and in most cases, a normal ICV occurs in trigonocephaly patients.

Furthermore, we found negligible correlation between the severity of the skull deformation and ICV, which shows that a more severe case of trigonocephaly does not give a larger difference in ICV than a mild trigonocephaly.

Elevated ICP is frequently seen in trigonocephaly patients and is caused by restricted skull growth. This may lead to ocular problems (papilledema, optic atrophy) found as part of routine screening of the ophthalmologist by fundoscopy, which is performed as part of screening prior to craniofacial surgery (Gault et al., 1990; Fok et al., 1992). The reported prevalence of papilledema in trigonocephaly patients is 1.5% and reported prevalence of raised ICP in trigonocephaly patients ranges from 8% to 20% in single-suture craniosynostosis (Gault et al., 1990; Renier et al., 1982; Eide et al., 2002; Tamburrini et al., 2005; Cornelissen et al., 2017). It should be noted that ICV measurement does not give (direct) information about ICP and the exact relationship between raised ICP and ICV remains unclear; however, in previous literature, no correlation between raised ICP and ICV was found (Gault et al., 1990; Fok et al., 1992). In our patient group, no papilledema was reported.

A limitation of the study is that the absence of papilledema in young children does not exclude the presence of elevated ICP; however, objectifying the ICP in young children is an invasive procedure and therefore the less invasive diagnostic fundoscopy is preferred as screening tool (Tuite et al., 1996). Furthermore, ICP monitoring is the diagnostic of choice; however, this technique is invasive.

In conclusion, we found normal preoperative values of ICV in patients with trigonocephaly compared to an age- and gender-matched control group. No correlation was found between severity of trigonocephaly and ICV in our sample size. No papilledema was reported. Skull growth from the patent sutures effectively compensates the decreased growth from the synostotic metopic suture as well in mild as in severe trigonocephaly cases.
